# Development and internal validation of a prediction model to identify older adults at risk of low physical activity levels during hospitalisation: a prospective cohort study

**DOI:** 10.1186/s12877-022-03146-9

**Published:** 2022-06-03

**Authors:** Hanneke C. van Dijk-Huisman, Mandy H. P. Welters, Wouter Bijnens, Sander M. J. van Kuijk, Fabienne J. H. Magdelijns, Robert A. de Bie, Antoine F. Lenssen

**Affiliations:** 1 Department of Physiotherapy, Maastricht University Medical Centre, P.O. Box 5800, 6202 AZ Maastricht, the Netherlands; 2 CAPHRI School for Public Health and Primary Care, Maastricht University, P.O. Box 616, 6200 MD Maastricht, the Netherlands; 3 Research Engineering (IDEE), Maastricht University, P.O. Box 616, 6200 MD Maastricht, The Netherlands; 4 Department of Clinical Epidemiology and Medical Technology Assessment, Maastricht University Medical Centre, P.O. Box 5800, 6202 AZ Maastricht, the Netherlands; 5 Department of Internal Medicine, Division of General Medicine and Clinical Geriatric Medicine, Maastricht University Medical Centre, P.O. Box 5800, 6202 AZ Maastricht, the Netherlands; 6 Department of Epidemiology, Maastricht University, P.O. Box 616, 6200 MD Maastricht, the Netherlands

**Keywords:** Physical activity, Hospital, Prediction model, Older adults

## Abstract

**Background:**

Inactive behaviour is common in older adults during hospitalisation and associated with poor health outcomes. If patients at high risk of spending little time standing/walking could be identified early after admission, they could be given interventions aimed at increasing their time spent standing/walking. This study aims to identify older adults at high risk of low physical activity (PA) levels during hospitalisation.

**Methods:**

Prospective cohort study of 165 older adults (≥ 70 years) admitted to the department of Internal Medicine of Maastricht University Medical Centre for acute medical illness. Two prediction models were developed to predict the probability of low PA levels during hospitalisation. Time spent standing/walking per day was measured with an accelerometer until discharge (≤ 12 days). The average time standing/walking per day between inclusion and discharge was dichotomized into low/high PA levels by dividing the cohort at the median (50.0%) in model 1, and lowest tertile (33.3%) in model 2. Potential predictors—Short Physical Performance Battery (SPPB), Activity Measure for Post-Acute Care (AM-PAC), age, sex, walking aid use, and disabilities in activities of daily living—were selected based on literature and analysed using logistic regression analysis. Models were internally validated using bootstrapping. Model performance was quantified using measures of discrimination (area under the receiver operating characteristic curve (AUC)) and calibration (Hosmer and Lemeshow (H–L) goodness-of-fit test and calibration plots).

**Results:**

Model 1 predicts a probability of spending ≤ 64.4 min standing/walking and holds the predictors SPPB, AM-PAC and sex. Model 2 predicts a probability of spending ≤ 47.2 min standing/walking and holds the predictors SPPB, AM-PAC, age and walking aid use. AUCs of models 1 and 2 were .80 (95% confidence interval (CI) = .73—.87) and .86 (95%CI = .79—.92), respectively, indicating good discriminative ability. Both models demonstrate near perfect calibration of the predicted probabilities and good overall performance, with model 2 performing slightly better.

**Conclusions:**

The developed and internally validated prediction models may enable clinicians to identify older adults at high risk of low PA levels during hospitalisation. External validation and determining the clinical impact are needed before applying the models in clinical practise.

**Supplementary Information:**

The online version contains supplementary material available at 10.1186/s12877-022-03146-9.

## Background

Older adults admitted to hospital with an acute medical illness show high prevalence of multimorbidity and age-related impairments, such as malnutrition, cognitive impairment, incontinence, and sensory impairment [1]. Combined with their decreased physiological and functional reserve capacity, this can result in poor outcomes [2–4]. Moreover, older adults spent little time standing and walking during hospitalisation. On average, between 30 and 117 min is spent standing or walking per day, the remainder of the day is spent lying in bed or sitting in a chair [5–12].

Inactive behaviour during hospitalisation is strongly associated with functional decline [2, 12–14], increased length of hospital stay [13], increased risk of institutionalization [2, 5, 15], and mortality [2, 6, 8, 16]. The association between physical inactivity and these negative outcomes is independent of illness severity or comorbidities [2, 6, 9, 14, 17, 18]. If patients at high risk of spending little time standing and walking could be identified early after admission, they could be given targeted interventions aimed at increasing their time spend standing and walking, such as guidance from a physiotherapist. As offering such interventions may require substantial resources, we aimed to be able to identify patients that are the least active and that are likely to benefit most from increasing their PA behaviour. Identification of these patients can therewith contribute to improved patient outcomes as well as value-based healthcare.

To our knowledge, a prognostic tool that predicts a patient’s probability of spending little time standing and walking during hospitalisation has not been developed yet. However, in recent years the number of studies investigating physical activity (PA) behaviour of older adults admitted to a hospital with an acute medical illness has grown and more insight has been gained in factors associated with inactive behaviour during hospitalisation [7–10, 14, 19–22]. Because of the association between inactive behaviour and functional decline we expect that functional assessment tools like the Short Physical Performance Battery (SPPB) and the Activity Measure for Post-Acute Care Inpatient Basic Mobility short form (AM-PAC) could help to accurately predict the probability of spending little time standing and walking during hospitalisation for older adults. Evensen et al. supported this by showing an association between SPPB-score and time spent standing and walking in older adults acutely admitted to a geriatric ward [9]. Moreover, age [7, 19], sex [19], disabilities in activities of daily living (ADL) two weeks preceding admission [7–10, 14, 19–21], and the use of a walking aid preceding hospitalisation [7, 20, 22] are also reported to be associated with patients’ PA behaviour during hospitalisation. Therefore, these factors may also contribute to predictive accuracy. The aim of this study is to develop and validate a prediction model that can be used early after admission to identify older adults at high risk of spending little time standing and walking during hospitalisation.

## Methods

### Study Design

This single centre, prospective cohort study was conducted at the department of Internal Medicine of Maastricht University Medical Centre (MUMC +) in Maastricht, the Netherlands, between October 2018 and March 2020. The Transparent Reporting of a multivariable prediction model for Individual Prognosis or Diagnosis (TRIPOD) statement was used as reporting guideline (Additional file 1) [23].

### Study Population

Older adults, admitted to the department of Internal Medicine of the MUMC + for acute care were recruited on weekdays by their attending physician and were asked for consent to be contacted by a researcher. Patients received verbal and written information about the study from the researcher within 48 h after admittance. The researcher contacted the patients again the next day, and written informed consent was obtained before study initiation. Confidentiality of data processing and anonymity of the participant were guaranteed.

Eligible patients were included when: 70 years or older, admitted to the department of Internal Medicine with an acute medical illness, sufficient understanding of the Dutch language, living at home before hospitalisation, and able to walk independently two weeks before admission as reported on the Functional Ambulation Categories (FAC > 3) [24, 25]. Exclusion criteria were: presence of contraindications to walking or wearing an accelerometer on the upper leg, mentally incapacitated subjects, inability to follow instructions due to cognitive problems or severe agitation, (re)admittance to the intensive care unit, a life expectancy of less than three months and previous participation in this study.

The following criteria were established through performing a brief screening with the attending physician prior to the informed consent procedure: age, admission for an acute medical illness, presence of contraindications to walking or wearing an accelerometer, mental incapacity, and life expectancy. Remaining criteria were checked by patient report.

### Procedure

#### Physical activity monitoring

PA monitoring started immediately after informed consent was obtained (t_0_). PA was monitored with the MOX activity monitor (MOX; Maastricht Instruments B.V., the Netherlands). The device contains a tri-axial accelerometer sensor (ADXL362; Analog Devices, Norwood, MA, USA) in a small waterproof housing (35 × 35 × 10 mm, 11 g). Raw acceleration data (± 8 g) were measured by three orthogonal sensor axes (X, Y and Z) at a 25 Hz sampling rate. PA was measured in time spent standing/walking as this was deemed a more appropriate sensor based outcome variable for hospitalised older adults than intensity levels or step counts[26]. The MOX activity monitor has been validated to differentiate lying/sitting from standing/walking in hospitalised patients [27]. A trained researcher fixated the accelerometer to the anterior thigh with a hypoallergenic plaster, 10 cm proximal of the patella. PA was continuously measured and each accelerometer was replaced with a fully charged one after seven days when needed. Nurses examined the skin for irritation every day. PA monitoring ended after twelve days or at the day of discharge, whichever came first. After removal of the accelerometer, raw accelerometer data was uploaded to a computer and participation in the study ended (t_1_).

A complete measurement day was defined as a 24-h interval starting and ending at midnight. If the accelerometer was temporarily removed (e.g., MRI), days with ≥ 20 h of wear time were included in the analysis. MATLAB (version 9.5 (R2018b) Natick, Massachusetts: The MathWorks Inc.: Natick, MA, USA; 2018) was used to calculate the number of minutes spent standing/walking per day. Subsequently, the average number of minutes spent standing/walking per day between t_0_ and t_1_ was calculated per patient.

For prediction model development, the average number of minutes spent standing/walking per day between t_0_ and t_1_ was dichotomized into low and high PA levels. Clinical guidelines stipulating the amount of time patients should to be standing/walking during hospitalisation do not exist yet [11, 21, 28–31]. Guidelines for healthy elderly exist, but are not suitable for hospitalised elderly [32–34]. As it was not possible to determine the optimal cut-off value for the dichotomization of time spent standing/walking based on existing recommendations, a data-driven approach was used with norm-referenced cut-off values instead of criterion-referenced cut-off values. Norm-referenced cut-off values were based on the prevalence of low PA levels (32%-50%) in previous studies [2, 14, 19, 35]. To enable the comparison of models with different cut-off values, two prediction models were developed with cut-off values capturing this range. For model 1, the average number of minutes spent standing/walking per day between t_0_ and t_1_ was dichotomized into low and high PA levels by dividing the cohort at the median, categorizing 50.0% of the patients as having low PA levels. For development of model 2, the cohort was divided at the lowest tertile, categorizing 33.3% of the patients as having low PA levels. The use of accelerometers allowed the assessment of low or high PA levels between t_0_ and t_1_ to remain blinded.

#### Potential predictors

Potential predictors were preselected based on published studies reporting factors associated with inactive behaviour of older adults admitted to a hospital with an acute medical illness [7–10, 14, 19, 20]. The following six predictors were preselected: SPPB, AM-PAC, age, sex, disabilities in ADL two weeks preceding admission, and walking aid use preceding hospitalisation.

Functional mobility was assessed by the researcher immediately after PA monitoring had started, using the SPPB and AM-PAC. The SPPB is a performance based tool to measure physical performance by assessing balance, walking speed and lower extremity strength [36, 37]. It provides a total score between 0 and 12 points, with 12 points reflecting the highest level of performance [36, 38]. The SPPB has good to excellent intra-rater and inter-rater reliability, and excellent criterion validity and responsiveness. It presents a good balance between mobility, measurement properties and applicability to an acute care geriatric unit [36].

 The AM-PAC Inpatient Basic Mobility short form assesses the following daily activities: turning in bed, sitting down and standing up, moving from lying to sitting, moving from a bed to chair, walking and climbing stairs. Climbing stairs was left out of the analysis as not every patient needs to climb stairs at home. This provided a total score between 1 and 20 points, dichotomized into dependent (≤ 19 points = 0) versus independent mobility (20 points = 1) based on receiver operating characteristic (ROC) curve analysis. The AM-PAC is short and easy to use. It shows large inter-rater reliability and test–retest reliability [39, 40] and has been validated for the entire hospital population [41].

 Disabilities in ADL two weeks preceding admission were reported on the Katz Index of Independence in Activities of Daily Living (Katz ADL) at t_0_. It rates the patient’s performance of six activities (bathing, dressing, toileting, transferring, continence, feeding) on a dichotomous scale (dependent/independent) [42, 43]. The number of disabilities was dichotomized (0/ ≥ 1 disabilities) based on ROC curve analysis. Although few reliability and validity studies exist, the Katz ADL is used extensively to assess functional capabilities of older adults in clinical settings [43, 44]. Furthermore, age, sex (0 = male/1 = female), and walking aid use preceding hospitalisation (none, walker, cane/crutch) were assessed by patient report at t_0_.

#### Medical and demographic data

At t_1_, the following data was extracted from the electronic health record: clinical diagnosis of the current hospitalisation based on the ICD-11[45], number of comorbidities (Charlson Comorbidity Index), experienced falls in the last six months (0/ ≥ 1 falls), physiotherapy consulted during hospitalisation (yes/no), length of stay in hospital (days) and discharge location (home, geriatric rehabilitation centre, nursing home, other).

### Sample Size

This study initially aimed to develop only one model, categorizing 50.0% of the patients as having low PA levels. Therefore, the sample size calculation was based on model 1. Post-hoc discussions regarding optimal cut-off values of low PA levels resulted in the development of a second model, enabling the comparison of different cut-off values. Six potential predictors were preselected. Because ‘walking aid use preceding hospitalisation’ has a categorical outcome containing three categories it had to be counted double, resulting in a sample size calculation based on seven potential predictors. It is recommended that at least 10 events should be collected per potential predictor [46]. An event is defined as the outcome status ‘low PA levels during hospitalisation’, with an estimated event rate of 50.0%. To develop a model with seven potential predictors, at least 70 events were required, resulting in a sample size of at least 140 patients (70/50*100). Based on the assumption of a 15% dropout rate, 165 patients were needed in this study.

### Data analysis

#### Data quality and missing data

Data were checked for completeness and inconsistencies. Any inconsistencies or incomplete data were corrected or completed. Missing values were imputed using stochastic regression imputation with fully conditional specification [47, 48]. To determine whether imputation led to radically different results, a sensitivity analysis was performed by comparing the outcomes of the imputed data set with the use of complete cases only.

#### Study population characteristics

Characteristics of patients were compared between the low and high PA level groups. To compare proportions, the chi-square test was used. For continuous variables, the independent samples t-test or Mann–Whitney U test was used for normally and not-normally distributed data, respectively. A *P*-value < 0.05 was used to indicate statistical significance.

#### Model development

The models were developed using ‘low PA levels during hospitalisation’ as the outcome variable. Multicollinearity of potential predictor variables was checked using collinearity diagnostics (Pearson correlation coefficients, variance inflation factor (VIF) and tolerance). Additionally, continuous variables were checked for having a linear association with the log odds of the outcome. For model 1, all seven predictors were introduced in a multivariable logistic regression model. For model 2, only five predictors could be introduced as the sample size was based on model 1 and the prevalence of low PA levels was lower in model 2. Therefore, univariable regression analysis was performed as additional step to select five potential predictors. To reduce the number of predictors in the multivariable logistic regression models, backward stepwise elimination based on the Wald test was used. A liberal *P-*value of 0.20 was used to prevent too early deletion of potentially relevant predictors [49].

#### Internal validation

The models were internally validated using bootstrapping. *B*-bootstrap samples of the same size as the original sample (*B* = 1000 was used) were drawn with replacement from the original data, reflecting the drawing of samples from the underlying population. A shrinkage factor was estimated to adjust the model coefficients in order to make future predictions less extreme. After shrinkage, the model intercepts were re-estimated to prevent systematic under- or overestimation of risks.

#### Performance of the model

Overall performance of both models was assessed using Nagelkerke's *R*^*2*^ and the Brier score. The ability of the models to discriminate between patients with low and high PA levels during hospitalisation was quantified as the area under the receiver operating characteristic curve (AUC). Additionally, sensitivity, specificity, positive predictive value (PPV), and negative predictive value (NPV) were calculated for a selection of probability cut-off values. To classify patients as being at high risk of low PA levels during hospitalisation, a probability threshold can be used. Patients are considered at high risk if their predicted probabilities are at or above this threshold. In order to have a low rate of patients misclassified as being at low risk (i.e. false-negative predictions), a probability threshold yielding a high NPV, but acceptable PPV, was chosen per model.

The agreement between predicted probabilities and observed frequencies of the outcome (accuracy) was assessed by visually inspecting the calibration plot. Furthermore, a Hosmer and Lemeshow (H–L) goodness-of-fit statistic was computed, with non-significant H–L statistics indicating good model fit. All statistical analyses were performed using SPSS version 23.0.0.2 (SPSS, Chicago, III., USA) and R version 4.0.4 (www.r-project.org).

## Results

### Study population characteristics

**Fig. 1:**
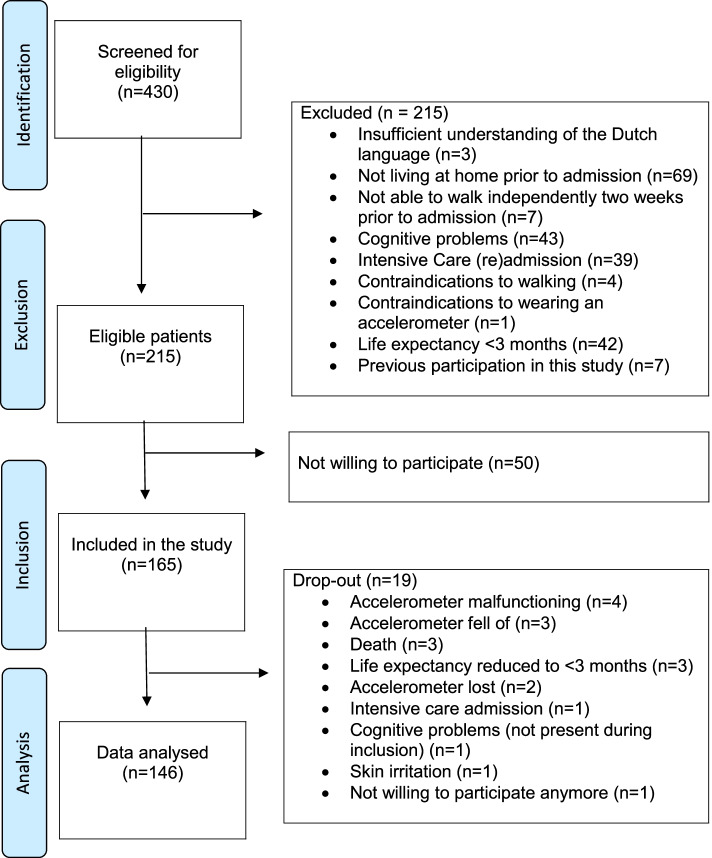
TRIPOD flow chart

Between October 2018 and March 2020, 430 older adults admitted with an acute medical illness were screened for eligibility. In total, 215 patients were identified as eligible and 165 patients were included in this study. Of the included patients, 19 (12%) dropped out and data of 146 patients was used in the analysis (Fig. 1 TRIPOD flow chart).

Of these 146 patients, the mean age (± standard deviation (SD)) was 81.3 (6.8) years and 86 (58.9%) patients were male. The group of patients were median (± Interquartile Range (IQR)) 64.4 (34.8 – 100.1) minutes standing/walking per day and 33.3% of the patients were ≤ 47.2 min standing/walking per day. The characteristics of study participants are reported in Table 1.

**Table 1:** Characteristics of study participants

		**Model 1**			**Model 2**		
**Variable**	**All patients** **(*n* = 146)**	**Low PA level (*n* = 73)**	**High PA level (*n* = 73)**	*p*-value*	**Low PA level (*n* = 49)**	**High PA level** **(*n* = 97)**	*p*-value*
Age, years (mean, SD)	81.3 (6.8)	82.4 (6.6)	80.2 (6.9)	.896	82.0 (6.8)	81.0 (6.8)	.363
Sex (n, %)				.737			.169
Female	60 (41.1%)	29 (39.7%)	31 (42.5%)		24 (49.0%)	36 (37.1%)	
Average min. standing/walking per day (median, IQR)	64.4 (34.7 – 100.1)	34.8 (16.9 – 51.8)	98.7 (78.1 –136.6)	< .001	24.4 (9.2 – 35.5)	84.6 (64.4 –124.9)	< .001
SPPB (median, IQR)	4 (2 – 8)	3 (1 – 5)	7 (4 – 10)	< .001	2 (0 – 3)	6 (4 – 10)	< .001
AM-PAC (n, %)				< .001			< .001
≤ 19	57 (39.0%)	44 (60.3%)	13 (17.8%)		36 (73.5%)	21 (21.6%)	
20	89 (61.0%)	29 (39.7%)	60 (82.2%)		13 (26.5%)	76 (78.4%)	
Katz ADL (n, %)				.045			< .001
0 disabilities	82 (56.2%)	35 (47.9%)	47 (64.4%)		17 (34.7%)	65 (67.0%)	
≥ 1 disabilities	64 (43.8%)	38 (52.1%)	26 (35.6%)		32 (65.3%)	32 (33.0%)	
Walking aid (n, %)				.038			.001
None	79 (54.1%)	32 (43.8%)	47 (64.4%)		16 (32.7%)	63 (64.9%)	
Walker	49 (33.6%)	31 (42.5%)	18 (24.7%)		26 (53.1%)	23 (23.7%)	
Crutch or cane	18 (12.3%)	10 (13.7%)	8 (11.0%)		7 (14.3%)	11 (11.3%)	
Clinical diagnosis (n, %)				.065			.120
Digestive	35 (24.0%)	15 (20.5%)	20 (27.4%)		9 (18.4%)	26 (26.8%)	
Respiratory	27 (18.5%)	10 (13.7%)	17 (23.3%)		7 (14.3%)	20 (20.6%)	
Infectious	23 (15.8%)	17 (23.3%)	6 (8.2%)		13 (26.5%)	10 (10.3%)	
Neoplasms	16 (11.0%)	10 (13.7%)	6 (8.2%)		7 (14.3%)	9 (9.3%)	
Genitourinary	14 (9.6%)	9 (12.3%)	5 (6.8%)		3 (6.1%)	11 (11.3%)	
Circulatory	7 (4.8%)	2 (2.7%)	5 (6.8%)		1 (2.0%)	6 (6.2%)	
Other	24 (16.4%)	10 (13.7%)	14 (19.2%)		9 (18.4%)	15 (15.5%)	
Comorbidities (CCI) (median, IQR)	2 (1 – 4)	3 (1 – 4)	2 (1 – 3)	.064	3 (1.5 – 5)	2 (1 – 3)	.001
Nr. of falls ≤ 6 months (median, IQR)	0 (0—1)	0 (0 – 2)	0 (0 – 1)	.386	0 (0 – 2)	0 (0 – 1)	.086
PT consulted (n, %)				< .001			< .001
Yes	89 (61.0%)	55 (75.3%)	34 (46.6%)		40 (81.6%)	49 (50.5%)	
No	57 (39.0%)	18 (24.7%)	39 (53.4%)		9 (18.4%)	48 (49.5%)	
LOS, days (median, IQR)	9 (6 – 13)	11 (7 – 15)	8 (6 – 11)	.001	13 (8 – 17)	8 (6 – 11)	< .001
Discharge location (n, %)				.001			< .001
Home	119 (81.5%)	50 (68.5%)	69 (94.5%)		29 (59.2%)	90 (92.8%)	
Geriatric rehabilitation centre	16 (11.0%)	13 (17.8%)	3 (4.1%)		12 (24.5%)	4 (4.1%)	
Nursing home	6 (4.1%)	5 (6.8%)	1 (1.4%)		3 (6.1%)	3 (3.1%)	
Other	5 (3.4%)	5 (6.8%)	0 (0.0%)		5 (10.2%)	0 (0.0%)	

Characteristics of study participants (older adults hospitalised with an acute medical illness) categorized by low or high PA levels, with cut-off values of 64.4 and 47.2 min standing/walking in model 1 and 2, respectively. *PA* Physical Activity, *SD* standard deviation, *IQR* Interquartile Range, *SPPB* Short Physical Performance Battery, *AM-PAC* Activity Measure for Post-Acute Care Inpatient Basic Mobility short form, *Katz ADL* Katz Index of Independence in Activities of Daily Living, *CCI* Charlson Comorbidity Index, *PT* physiotherapy, *LOS* length of hospital stay

^*^P-value < 0.05. To compare proportions, the chi-square test was used. For continuous variables, the independent sample t-test or Mann–Whitney U test were used for normally and not-normally distributed data, respectively

### Model development and internal validation

In the dependent variable ‘number of minutes spent standing/walking per day’, data was missing for 72 out of 949 measurement days (7.5%), spread over 27 patients (18%). Main reasons for missing values were the accelerometer falling off, getting lost or malfunctioning. Data of all other variables was complete. After imputation, data of all 146 patients was complete for development of the prediction model.

For development of model 1, all predictors were entered in the multivariable regression model (SPPB, AM-PAC, age, sex, disabilities in ADL, and walking aid use). For development of model 2, univariable regression analysis was first performed on all potential predictors, after which SPPB, AM-PAC, age, and walking aid use preceding hospitalisation were entered in the multivariable regression model. Table 2 shows the original and internally validated models that can be used to compute the probability of low PA levels during hospitalisation. Internal validation yielded a shrinkage factor of 0.95 and 0.90 in model 1 and 2, respectively. The equations in Table 2 can be used to compute the individual probability of low PA levels during hospitalisation.

**Table 2:** Regression coefficients and odds ratios with 95% CI from the original and internally validated models

	**Original Model**			**Model after internal validation**
**Variable**	**Regression coefficient**	**Odds Ratio (95% CI)**	***p*-value**	**Regression coefficient**^a^
**Model 1**—Cut-off value for low or high PA levels at 50.0% of the cohort (64.4 min standing/walking)				
Intercept	2.042	-	.000	1.942
SPPB	-.251	.778 (.677 – .894)	.000	-.239
AM-PAC (independent)	-.894	.409 (.159 – 1.054)	.064	-.850
Sex (female)	-.519	.595 (.269 – 1.313)	.199	-.493
**Model 2**—Cut-off value for low or high PA levels at 33.3% of the cohort (47.2 min standing/walking)				
Intercept	7.008	-	.022	6.255
SPPB	-.305	.737 (.608 – .894)	.002	-.275
AM-PAC (independent)	-1.124	.325 (.115 – .921)	.034	-1.012
Age	-.078	.925 (.861 – .994)	.034	-.070
Walking aid				
Crutch/Cane	-.006	.994 (.248 – 3.977)	.993	-.006
Walker	1.281	3.601 (1.317 – 9.843)	.013	1.153
To estimate the individual probability of low PA levels during hospitalisation: ** Model 1:** P_(Low PA)_ = 1 / (1 + e^(−(1.942 − .239*SPPB − .850*AM−PAC − .493*Female))^) ^*^100% ** Model 2:** P_(Low PA)_ = 1 / (1 + e^(−(6.255 − .275*SPPB − 1.012*AM−PAC − .070*Age − .006*Crutch/Cane + 1.153*Walker))^) ^*^100%				

*CI* confidence interval, PA Physical Activity, SPPB Short Physical Performance Battery, AM-PAC Activity Measure for Post-Acute Care Inpatient Basic Mobility short form

^a^Regression coefficients after adjustment for overfitting by shrinkage (shrinkage factor model 1 = 0.95 and model 2 = 0.90); the intercept was re-estimated

### Performance of the models

**Fig. 2:**
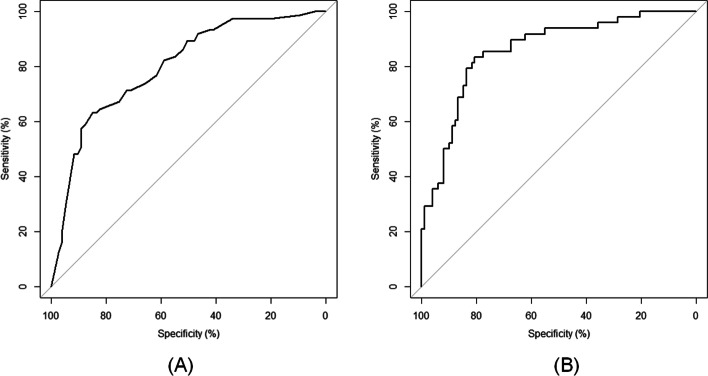
Receiver operating characteristic curve of (**A**) model 1 (AUC .80 (95% confidence interval (CI) = .73—.87)), and (**B**) model 2 (AUC .86 (95%CI = .79—.92))

All model performance measures are shown in detail in Additional file 2. The AUCs of model 1 and 2 were 0.80 (95% confidence interval (CI) = 0.73—0.87) and 0.86 (95%CI = 0.79—0.92), respectively, indicating good discriminatory ability. The optimism-corrected AUCs were 0.79 and 0.84 for model 1 and 2, respectively. Figure 2 shows the ROC curves of both models. Both models show good calibration, as indicated by calibration plots showing good agreement between actual and predicted probabilities (Additional file 3). Additionally, both H–L goodness-of-fit tests were non-significant (*p* = 0.755 and *p* = 0.209). Overall, model 2 showed a slightly better performance.

**Fig. 3:**
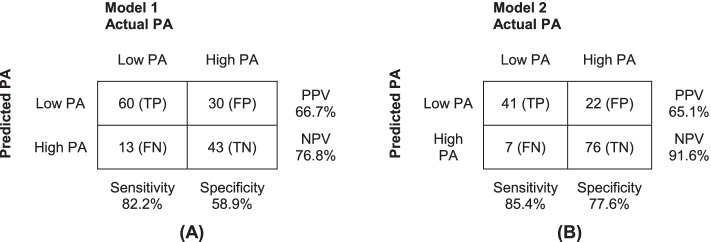
Classification tables showing the actual and predicted number of patients with low or high PA levels during hospitalisation and their corresponding sensitivity, specificity, PPV and NPV, using (**A**) model 1 (probability threshold .41) and (**B**) model 2 (probability threshold .30). PA = Physical Activity, TP = True Positive, FP = False Positive, FN = False Negative, TN = True Negative, PPV = Positive Predictive Value, NPV = Negative Predictive Value

A selection of probability cut-off values and their corresponding sensitivity, specificity, PPV and NPV are reported in Additional file 4 and Additional file 5. Probability thresholds of 0.41 and 0.30 were chosen for model 1 and 2, respectively. The corresponding classification tables are shown in Fig. 3.

The sensitivity analyses showed similar results for the imputed and the original non-imputed data sets and we conclude that imputation did not result in large differences.

## Discussion

In this study we developed and internally validated two prediction models that can be used to predict the probability of low PA levels during hospitalisation for older adults admitted to a hospital with an acute medical illness. The first model predicts a patient’s probability of spending less than an average of 64.4 min standing/walking per day and holds three predictors: SPPB, AM-PAC and sex. The second model predicts a patient’s probability of spending less than an average of 47.2 min standing/walking per day and holds four predictors: SPPB, AM-PAC, age and walking aid use preceding hospitalisation. Both models showed good discriminative ability and accurate prediction of spending little time standing/walking during hospitalisation, with the second model performing slightly better.

To our knowledge, this is the first study that aims to identify older adults at high risk of spending little time standing/walking during hospitalisation. One of the challenges in developing a suitable prediction model was the lack of criterion-referenced cut-off values regarding the classification of low or high PA levels [11, 21, 28–31]. Although many studies have shown that PA contributes to the prevention of negative outcomes, the optimal-dose response relationship remains unknown. Baldwin et al. provided the first international consensus for recommendations on PA and sedentary behaviour for older adults hospitalised with an acute medical illness. They recommend that older adults should: be as physically active as their abilities and condition allow; minimise time spent sedentary for extended periods; and, move more and sit less throughout the day. Additionally, muscle strengthening and balance exercises are also advised [29]. Although this provides some guidance, criterion-referenced cut-off values regarding the classification of low and high PA levels are still lacking [11, 21, 28–31]. Therefore, the current prediction models were developed using a data-driven approach with norm-referenced cut-off values capturing the range of low PA levels identified in previous studies [2, 14, 19, 35]. We do not know whether the resulting cut-off values of 64.4 or 47.2 min standing/walking are sufficient to prevent the negative effects of inactivity. This may also be influenced by many individual factors such as preadmission status, illness severity or daily caloric intake, necessitating more personalised recommendations. However, as the PA behaviour of older adults admitted for acute illness was heterogeneous in previous literature, we aimed to be able to identify patients that are the least active. As offering interventions (e.g., monitoring patients’ PA behaviour using wearables) may require substantial financial resources, the prediction models allow to identify patients that are likely to benefit most from such interventions. Although criterion-referenced cut-off values are lacking, the chosen cut-off values are relevant and contribute to providing value-based healthcare.

PA was measured in time spent in different activities (lying/sitting, standing/walking), as this was deemed a more meaningful sensor based outcome variable for hospitalised older adults than intensity levels (activity counts) or step counts [26]. First, the intensity of PA as perceived by patients may deviate from the intensity measured by the accelerometer. When patients are feeling ill they may perceive walking at low walking speeds as a high intensity activity, while the accelerometer objectively classifies this as a low intensity activity. Second, recovery or deterioration during the admission period may result in fluctuations in perceived intensity within patients. Third, many older adults admitted to hospital with an acute medical illness require a walking aid. Moreover, slow and impaired gaits are common [50]. Several studies have shown that these factors decrease the validity of activity trackers to measure step counts [51–54]. Lastly, movements of the arms or legs performed in bed or on a chair may result in an overestimation of step count. Therefore, time spent in different activities is deemed the most appropriate sensor-based outcome measure [26].

The developed models show that functional assessments combined with easily acquired clinical parameters have potential to identify patients at high risk of low PA levels during hospitalisation. As we felt it important that patients at high risk would not miss out on an intervention, we opted for models with low rates of false-negative predictions. In choosing a probability threshold for each model we therefore aimed for a high NPV and accepted a higher rate of false-positive predictions.

Using model 1 with a probability threshold of 0.41 resulted in misclassification of 8.9% of all patients as being at low risk of spending less than 64.4 min standing/walking (false-negative predictions). Moreover, 20.6% were misclassified as being at high risk (false-positive predictions) and would be given an intervention while actually having high PA levels. The predictive abilities of model 2 are slightly better, resulting in less misclassifications. Using model 2 with a probability threshold of 0.30 will result in a 4.8% false-negative prediction rate and a 15.1% false-positive prediction rate of spending less than 47.2 min standing/walking.

However, a certain level of misclassifications seems inevitable when predicting the PA behaviour of hospitalised patients early after admission. Previous studies have shown that the PA behaviour of hospitalised patients is influenced by many different factors, such as complications or symptoms developing throughout the hospital stay, patient motivation, using medical devices that limit walking (e.g., IV-poles, urinary catheters, lack of portable oxygen), referral to physiotherapy, or a lack of availability of healthcare staff to provide assistance during walking [55–61]. As some of these factors are unknown yet early after admission, they cannot be included in the models as predictors and may therefore contribute to misclassifications.

In retrospect, we consider the rate of misclassifications of both models acceptable for use in clinical practice. Although performance of model 2 was slightly better, the choice for either one of the models depends on whether roughly one hour or three quarters of an hour standing/walking are preferred as cut-off value for low PA levels during hospitalisation. Moreover, it also depends on the availability of resources, as model 1 will classify more patients as being at high risk of low PA levels.

### Strengths and limitations

The present study has several strengths, including a prospective data collection, recruitment of consecutive patients, and inclusion of patients with mild cognitive impairments. Moreover, all predictors were selected from literature based on previous evidence [7–10, 14, 19, 20] while the methodology of this study followed the TRIPOD guideline for prediction modelling [23]. We corrected for missing data, performed a sensitivity analysis and an internal validation procedure of the developed models.

We also recognise several limitations. First, with the current study design we cannot determine if patients changed their time spent standing/walking due to potential confounding factors that were unknown early after admission, such as receiving physiotherapy guidance or complications which may have developed during hospital stay. Second, we initially aimed to develop one prediction model. Due to post-hoc discussions regarding an optimal cut-off value we decided to develop a second model as well, enabling the comparison of different cut-off values. The power calculation was originally meant for model 1, allowing for seven potential predictors and an event rate of 50.0%. Because the event rate of model 2 was 33.3%, only five potential predictors could be introduced in the analysis. To prevent overfitting of the model by introducing to many predictors, univariable analysis had to be performed as an additional step. Third, we dichotomized the categorical AM-PAC and Katz ADL outcomes to improve the clinical applicability, which may have led to loss of information. Lastly, the algorithm of the MOX activity monitor is unable to differentiate time spent standing from time spent walking. This limited the development of a prediction model that can be used to identify older adults at high risk of spending little time walking during hospitalisation.

### Clinical implications and recommendations for future research

The developed prediction models can be used in clinical practice by performing a simple screening early after admission, consisting of two functional assessments combined with self-reported information. The prediction models can be adapted into an easy-to-use calculator that can be used during screening. Using the prediction models to identify patients at high risk of low PA levels early after admission is an important first step in preventing the negative effects associated with spending little time standing/walking during hospitalisation. Patients at high risk can subsequently be given interventions aimed at increasing their time spend standing/walking. However, as few studies have investigated the efficacy of interventions aimed at increasing the PA behaviour of older adults during hospitalisation, further research is advised, comparing different types of interventions and with detailed reporting of frequency, intensity and duration [4, 29–31]. Moreover, the accelerometer algorithm used in the current study was not able to differentiate standing from walking. Therefore, we recommend future studies to develop a prediction model using an optimised accelerometer algorithm that allows to differentiate between standing and walking in hospitalised patients. Furthermore, the current study included seven potential predictors that were associated with low PA levels of older adults admitted to hospital with an acute medical illness. However, the PA behaviour of hospitalised older adults may be influenced by many other factors as well, such as ‘reason for hospitalisation’ or ‘history of falls’ [7, 20–22, 55–61]. In order to improve the prediction of older adults at risk of low PA levels, we recommend that this study should be followed by a larger study that allows to include more potential predictor variables. Lastly, before implementing the prediction models into clinical practice, future research should also focus on assessing the next steps within prediction modelling: determining the external validity and clinical impact of the models. Because this has not been performed yet in the current study, our results should be interpreted with caution. In order to choose a cut-off value for low PA levels during hospitalisation based on empirical evidence, future research should also focus on developing guidelines regarding the recommended PA levels of older adults admitted to a hospital with an acute medical illness.

## Conclusions

We developed and internally validated two prediction models that can be used to predict the probability of low PA levels during hospitalisation for older adults admitted to a hospital with an acute medical illness. Both models showed a good overall performance, with a good discriminative ability and accurate prediction of low PA levels. This study showed that both models hold promise as prediction tools that enable clinicians to accurately identify older adults at high risk of low PA levels during hospitalisation.

## Supplementary Information

**Additional file 1.** TRIPOD checklistPrediction Model Development. Checklist for transparent reporting of amultivariable prediction model for individual prognosis or diagnosis (TRIPOD):The TRIPOD statement.

**Additional file 2.** Performance of theprediction models. Model performance measures of the two prediction models forpredicting the probability of low physical activity levels duringhospitalisation for older adults admitted to a hospital with an acute medicalillness.

**Additional file 3.** Calibration plots. Calibrationplots with the observed frequency of low physical activity levels by predictedprobability of model 1 and 2.

**Additional file 4.** Probability cut-offvalues model 1. Sensitivity, specificity, positive predictive value, andnegative predictive value for low physical activity levels duringhospitalisation at a selection of consecutive cut-off points of the predictedprobability of model 1.

**Additional file 5.** Probability cut-offvalues model 2. Sensitivity, specificity, positive predictive value, andnegative predictive value for low physical activity levels duringhospitalisation at a selection of consecutive cut-off points of the predictedprobability of model 2.

## Data Availability

The datasets used and/or analysed during the current study are available from the corresponding author on reasonable request.

## Abbreviations

PA: Physical Activity
SPPB: Short Physical Performance Battery
AM-PAC: Activity Measure for Post-Acute Care Inpatient Basic Mobility short form
ADL: Activities of Daily Living
MUMC +: Maastricht University Medical Centre
TRIPOD: Transparent Reporting of a multivariable prediction model for Individual Prognosis or Diagnosis
FAC: Functional Ambulation Categories
MOX: MOX Activity Monitor
ROC: Receiver Operating Characteristic
ICD: International Classification of Diseases for mortality and morbidity
VIF: Variance Inflation Factor
AUC: Area Under the receiver operating characteristic Curve
PPV: Positive Predictive Value
NPV: Negative Predictive Value
H–L: Hosmer and Lemeshow
SD: Standard Deviation
IQR: Interquartile Range
CCI: Charlson Comorbidity Index
LOS: Length Of hospital Stay
CI: Confidence Interval
