# Supplementary material for: Development and internal validation of a prediction model to identify older adults at risk of low physical activity levels during hospitalisation: a prospective cohort study
Source: BMC Geriatr. 2022 Jun 3;22:479. doi: 10.1186/s12877-022-03146-9 (PMC9164480; doi:10.1186/s12877-022-03146-9)
Supplement: Supplementary file 2 — Additional file 2. Performance of theprediction models. Model performance measures of the two prediction models forpredicting the probability of low physical activity levels duringhospitalisation for older adults admitted to a hospital with an acute medicalillness. [file 12877_2022_3146_MOESM2_ESM.docx]

**Additional file 2.** Performance of the prediction models

| **Performance** | **Model 1** | **Model 2** |
| --- | --- | --- |
| Nagelkerke’s R^2^ | .34 | .45 |
| Brier score | .18 | .14 |
| AUC (95% CI) | .80 (.73 - .87) | .86 (.79- .92) |
| Optimism in AUC | .01 | .02 |
| Optimism-corrected AUC | .79 | .84 |
| H-L goodness-of-fit test (p-value) | .76 | .21 |
| Predicted probabilities (range) | .11 - .89 | .02 - .87 |
| Predicted probabilities (median, IQR) | .49 (.24 - .78) | .24 (.06 - .62) |

Model performance measures of the two prediction models for predicting the probability of low physical activity levels during hospitalisation for older adults admitted to a hospital with an acute medical illness.
AUC = area under the receiver operating characteristic curve, CI = confidence interval, H-L goodness-of-fit test = Hosmer-Lemeshow goodness-of-fit test, IQR = interquartile range
