# Supplementary material for: Development and internal validation of a prediction model to identify older adults at risk of low physical activity levels during hospitalisation: a prospective cohort study
Source: BMC Geriatr. 2022 Jun 3;22:479. doi: 10.1186/s12877-022-03146-9 (PMC9164480; doi:10.1186/s12877-022-03146-9)
Supplement: Supplementary file 3 — Additional file 3. Calibration plots. Calibrationplots with the observed frequency of low physical activity levels by predictedprobability of model 1 and 2. [file 12877_2022_3146_MOESM3_ESM.docx]

**Additional file 3.** Calibration plots


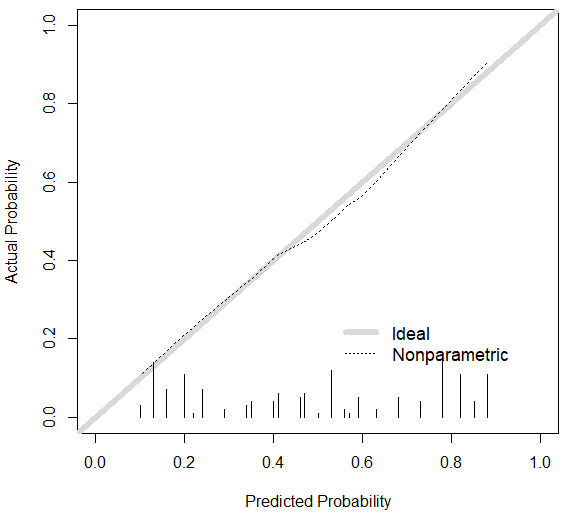

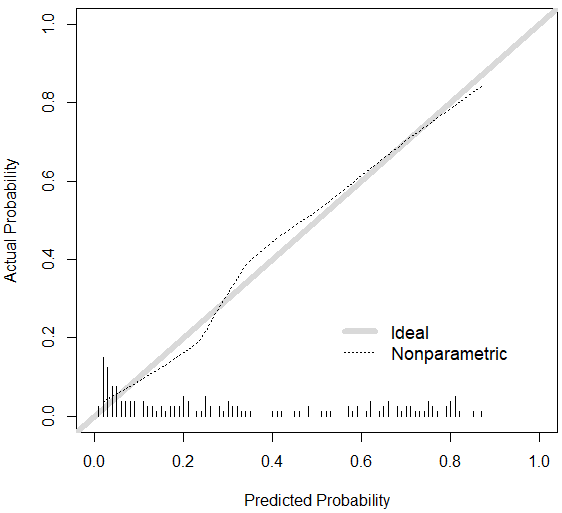


**(A) (B)**

Calibration plots with the observed frequency of low physical activity levels by predicted probability of **A)** model 1, and **B)** model 2.
