# Supplementary material for: Development and internal validation of a prediction model to identify older adults at risk of low physical activity levels during hospitalisation: a prospective cohort study
Source: BMC Geriatr. 2022 Jun 3;22:479. doi: 10.1186/s12877-022-03146-9 (PMC9164480; doi:10.1186/s12877-022-03146-9)
Supplement: Supplementary file 4 — Additional file 4. Probability cut-offvalues model 1. Sensitivity, specificity, positive predictive value, andnegative predictive value for low physical activity levels duringhospitalisation at a selection of consecutive cut-off points of the predictedprobability of model 1. [file 12877_2022_3146_MOESM4_ESM.docx]

**Additional file 4.** Probability cut-off values model 1

| **Predicted probability *** | **Sensitivity (%)** | **Specificity (%)** | **PPV (%)** | **NPV (%)** |
| --- | --- | --- | --- | --- |
| 0,12 | 100 (73/73) | 4,1 (3/73) | 51,0 (73/143) | 100,0 (3/3) |
| 0,13 | 98,6 (72/73) | 9,6 (7/73) | 52,2 (72/138) | 87,3 (7/8) |
| 0,15 | 97,3 (71/73) | 20,5 (15/73) | 55,0 (71/129) | 88,4 (15/17) |
| 0,16 | 97,3 (71/73) | 23,3 (17/73) | 55,9 (71/127) | 89,6 (17/19) |
| 0,18 | 97,3 (71/73) | 30,1 (22/73) | 58,2 (71/122) | 91,8 (22/24) |
| 0,20 | 97,3 (71/73) | 34,2 (25/73) | 59,7 (71/119) | 92,7 (25/27) |
| 0,21 | 93,2 (68/73) | 41,1 (30/73) | 61,3 (68/111) | 85,8 (30/35) |
| 0,23 | 93,2 (68/73) | 42,5 (31/73) | 61,8 (68/110) | 86,2 (31/36) |
| 0,25 | 91,8 (67/73) | 46,6 (34/73) | 63,2 (67/106) | 85,0 (34/40) |
| 0,27 | 89,0 (65/73) | 47,9 (35/73) | 63,1 (65/103) | 81,3 (35/43) |
| 0,30 | 89,0 (65/73) | 49,3 (36/73) | 63,7 (65/102) | 81,8 (36/44) |
| 0,32 | 89,0 (65/73) | 50,7 (37/73) | 64,4 (65/101) | 82,2 (37/45) |
| 0,35 | 86,3 (63/73) | 52,1 (38/73) | 64,3 (63/98) | 79,2 (38/48) |
| 0,38 | 83,6 (61/73) | 54,8 (40/73) | 64,9 (61/94) | 77,0 (40/52) |
| **0,41**** | **82,2 (60/73)** | **58,9 (43/73)** | **66,7 (60/90)** | **76,8 (43/56)** |
| 0,44 | 76,7 (56/73) | 61,6 (45/73) | 66,6 (56/84) | 72,6 (45/62) |
| 0,47 | 74,0 (54/73) | 65,8 (48/73) | 68,4 (54/79) | 71,7 (48/67) |
| 0,49 | 71,2 (52/73) | 71,2 (52/73) | 71,2 (52/73) | 71,2 (52/73) |
| 0,52 | 71,2 (52/73) | 72,6 (53/73) | 72,2 (52/72) | 71,6 (53/74) |
| 0,53 | 67,1 (49/73) | 75,3 (55/73) | 73,1 (49/67) | 69,6 (55/79) |
| 0,55 | 64,4 (47/73) | 82,2 (60/73) | 78,3 (47/60) | 69,8 (60/86) |
| 0,57 | 63,0 (46/73) | 83,6 (61/73) | 79,3 (46/58) | 69,3 (61/88) |
| 0,58 | 63,0 (46/73) | 84,9 (62/73) | 80,7 (46/57) | 69,6 (62/89) |
| 0,61 | 58,9 (43/73) | 87,7 (64/73) | 82,7 (43/52) | 68,1 (64/94) |
| 0,66 | 57,5 (42/73) | 89,0 (65/73) | 83,9 (42/50) | 67,7 (65/96) |
| 0,69 | 53,4 (39/73) | 89,0 (65/73) | 82,9 (39/47) | 65,6 (65/99) |
| 0,71 | 50,7 (37/73) | 89,0 (65/73) | 82,2 (37/45) | 64,4 (65/101) |
| 0,74 | 47,9 (35/73) | 90,4 (66/73) | 83,3 (35/42) | 63,4 (66/104) |
| 0,76 | 47,9 (35/73) | 91,8 (67/73) | 85,4 (35/41) | 63,8 (67/105) |
| 0,78 | 39,7 (29/73) | 93,2 (68/73) | 85,4 (29/34) | 60,7 (68/112) |
| 0,80 | 30,1 (22/73) | 94,5 (69/73) | 84,6 (22/26) | 57,5 (69/120) |
| 0,82 | 20,5 (15/73) | 95,9 (70/73) | 83,3 (15/18) | 54,7 (70/128) |
| 0,84 | 16,4 (12/73) | 95,9 (70/73) | 80,0 (12/15) | 53,4 (70/131) |
| 0,87 | 12,3 (9/73) | 97,3 (71/73) | 82,0 (9/11) | 52,6 (71/135) |

Sensitivity, specificity, PPV and NPV for low physical activity levels during hospitalisation at a selection of consecutive cut-off points of the predicted probability of model 1

PPV = Positive Predictive Value, NPV = Negative Predictive Value

* Patients are considered at high risk of low physical activity levels if their predicted probabilities are at or above this threshold.
** Suggested probability threshold for model 1.
