# Supplementary material for: Development and internal validation of a prediction model to identify older adults at risk of low physical activity levels during hospitalisation: a prospective cohort study
Source: BMC Geriatr. 2022 Jun 3;22:479. doi: 10.1186/s12877-022-03146-9 (PMC9164480; doi:10.1186/s12877-022-03146-9)
Supplement: Supplementary file 5 — Additional file 5. Probability cut-offvalues model 2. Sensitivity, specificity, positive predictive value, andnegative predictive value for low physical activity levels duringhospitalisation at a selection of consecutive cut-off points of the predictedprobability of model 2. [file 12877_2022_3146_MOESM5_ESM.docx]

**Additional file 5.** Probability cut-off values model 2

| **Predicted probability*** | **Sensitivity (%)** | **Specificity (%)** | **PPV (%)** | **NPV (%)** |
| --- | --- | --- | --- | --- |
| 0,02 | 100,0 (48/48) | 1,0 (1/98) | 33,1 (48/145) | 100,0 (1/1) |
| 0,03 | 100,0 (48/48) | 14,3 (14/98) | 36,4 (48/132) | 100,0 (14/14) |
| 0,04 | 97,9 (47/48) | 22,4 (22/98) | 38,2 (47/123) | 95,7 (22/23) |
| 0,05 | 95,8 (46/48) | 29,6 (29/98) | 40,0 (46/115) | 93,5 (29/31) |
| 0,06 | 95,8 (46/48) | 34,7 (34/98) | 41,8 (46/110) | 94,4 (34/36) |
| 0,07 | 93,8 (45/48) | 36,7 (36/98) | 42,1 (45/107) | 92,3 (36/39) |
| 0,08 | 93,8 (45/48) | 39,8 (39/98) | 43,3 (45/104) | 92,9 (39/42) |
| 0,09 | 93,8 (45/48) | 42,9 (42/98) | 44,6 (45/101) | 93,3 (42/45) |
| 0,10 | 93,8 (45/48) | 44,9 (44/98) | 45,5 (45/99) | 93,6 (44/47) |
| 0,12 | 93,8 (45/48) | 48,0 (47/98) | 46,9 (45/96) | 94,0 (47/50) |
| 0,13 | 93,8 (45/48) | 51,0 (50/98) | 48,4 (45/93) | 94,3 (50/53) |
| 0,14 | 93,8 (45/48) | 53,1 (52/98) | 49,5 (45/91) | 94,5 (52/55) |
| 0,15 | 93,8 (45/48) | 54,1 (53/98) | 50,0 (45/90) | 94,6 (53/56) |
| 0,16 | 91,7 (44/48) | 55,1 (54/98) | 50,0 (44/88) | 93,1 (54/58) |
| 0,17 | 91,7 (44/48) | 56,1 (55/98) | 50,6 (44/87) | 93,2 (55/59) |
| 0,18 | 91,7 (44/48) | 58,2 (57/98) | 51,8 (44/85) | 93,4 (57/61) |
| 0,19 | 91,7 (44/48) | 60,2 (59/98) | 53,0 (44/83) | 93,7 (59/63) |
| 0,20 | 89,6 (43/48) | 62,2 (61/98) | 53,8 (43/80) | 92,4 (61/66) |
| 0,21 | 89,6 (43/48) | 65,3 (64/98) | 55,8 (43/77) | 92,8 (64/69) |
| 0,22 | 89,6 (43/48) | 67,3 (66/98) | 57,3 (43/75) | 93,0 (66/71) |
| 0,23 | 87,5 (42/48) | 67,3 (66/98) | 56,8 (42/74) | 91,7 (66/72) |
| 0,24 | 85,4 (41/48) | 67,3 (66/98) | 56,2 (41/73) | 90,4 (66/73) |
| 0,25 | 85,4 (41/48) | 68,4 (67/98) | 56,9 (41/72) | 90,5 (67/74) |
| 0,26 | 85,4 (41/48) | 72,4 (71/98) | 60,3 (41/68) | 91,0 (71/78) |
| 0,27 | 85,4 (41/48) | 73,5 (72/98) | 61,2 (41/67) | 91,1 (72/79) |
| 0,28 | 85,4 (41/48) | 74,5 (73/98) | 62,1 (41/66) | 91,3 (73/80) |
| 0,29 | 85,4 (41/48) | 76,5 (75/98) | 64,1 (41/64) | 91,5 (75/82) |
| **0,30**** | **85,4(41/48)** | **77,6 (76/98)** | **65,1 (41/63)** | **91,6 (76/83)** |
| 0,31 | 83,3 (40/48) | 79,6 (78/98) | 66,7 (40/60) | 90,7 (78/86) |
| 0,32 | 81,3 (39/48) | 80,6 (79/98) | 67,2 (39/58) | 89,8 (79/88) |
| 0,33 | 79,2 (38/48) | 81,6 (80/98) | 67,9 (38/56) | 88,9 (80/90) |
| 0,34 | 79,2 (38/48) | 82,7 (81/98) | 69,1 (38/55) | 89,0 (81/91) |
| 0,35 | 79,2 (38/48) | 83,7 (82/98) | 70,4 (38/54) | 89,1 (82/92) |
| 0,38 | 77,1 (37/48) | 83,7 (82/98) | 69,8 (37/53) | 88,2 (82/93) |
| 0,41 | 75,0 (36/48) | 83,7 (82/98) | 69,2 (36/52) | 87,2 (82/94) |
| 0,42 | 72,9 (35/48) | 83,7 (82/98) | 68,6 (35/51) | 86,3 (82/95) |
| 0,44 | 72,9 (35/48) | 84,7 (83/98) | 70,0 (35/50) | 86,5 (83/96) |
| 0,46 | 70,8 (34/48) | 84,7 (83/98) | 69,4 (34/49) | 85,6 (83/97) |
| 0,48 | 68,8 (33/48) | 84,7 (83/98) | 68,8 (33/48) | 84,7 (83/98) |
| 0,49 | 68,8 (33/48) | 85,7 (84/98) | 70,2 (33/47) | 84,8 (84/99) |
| 0,50 | 68,8 (33/48) | 86,7 (85/98) | 71,7 (33/46) | 85,0 (85/100) |
| 0,52 | 66,7 (32/48) | 86,7 (85/98) | 71,1 (32/45) | 84,2 (85/101) |
| 0,53 | 64,6 (31/48) | 86,7 (85/98) | 70,5 (31/44) | 83,3 (85/102) |
| 0,55 | 62,5 (30/48) | 86,7 (85/98) | 69,8 (30/43) | 82,5 (85/103) |
| 0,58 | 60,4 (29/48) | 86,7 (85/98) | 69,0 (29/42) | 81,7 (85/104) |
| 0,59 | 58,3 (28/48) | 87,8 (86/98) | 70,0 (28/40) | 81,1 (86/106) |
| 0,61 | 56,3 (27/48) | 88,8 (87/98) | 71,1 (27/38) | 80,6 (87/108) |
| 0,62 | 54,2 (26/48) | 88,8 (87/98) | 70,3 (26/37) | 79,8 (87/109) |
| 0,63 | 50,0 (24/48) | 89,8 (88/98) | 70,6 (24/34) | 78,6 (88/112) |
| 0,65 | 50,0 (24/48) | 90,8 (89/98) | 72,7 (24/33) | 78,8 (89/113) |
| 0,66 | 47,9 (23/48) | 91,8 (90/98) | 74,2 (23/31) | 78,3 (90/115) |
| 0,67 | 43,8 (21/48) | 91,8 (90/98) | 72,4 (21/29) | 76,9 (90/117) |
| 0,68 | 41,7 (20/48) | 91,8 (90/98) | 71,4 (20/28) | 76,3 (90/118) |
| 0,69 | 37,5 (18/48) | 91,8 (90/98) | 69,2 (18/26) | 75,0 (90/120) |
| 0,70 | 37,5 (18/48) | 92,9 (91/98) | 72,0 (18/25) | 75,2 (91/121) |
| 0,71 | 35,4 (17/48) | 93,9 (92/98) | 73,9 (17/23) | 74,8 (92/123) |
| 0,72 | 35,4 (17/48) | 94,9 (93/98) | 77,3 (17/22) | 75,0 (93/124) |
| 0,73 | 33,3 (16/48) | 95,9 (94/98) | 80,0 (16/20) | 74,6 (94/126) |
| 0,74 | 31,3 (15/48) | 95,9 (94/98) | 78,9 (15/19) | 74,0 (94/127) |
| 0,75 | 29,2 (14/48) | 95,9 (94/98) | 77,8 (14/18) | 73,4 (94/128) |
| 0,76 | 29,2 (14/48) | 99,0 (97/98) | 93,3 (14/15) | 74,0 (97/131) |
| 0,77 | 25,0 (12/48) | 99,0 (97/98) | 92,3 (12/13) | 72,9 (97/133) |
| 0,78 | 22,9 (11/48) | 99,0 (97/98) | 91,7 (11/12) | 72,4 (97/134) |
| 0,80 | 20,8 (10/48) | 100,0 (98/98) | 100,0 (10/10) | 72,1 (98/136) |
| 0,81 | 14,6 (7/48) | 100,0 (98/98) | 100,0 (7/7) | 70,5 (98/139) |
| 0,82 | 6,3 (3/48) | 100,0 (98/98) | 100,0 (3/3) | 68,5 (98/143) |
| 0,84 | 4,2 (2/48) | 100,0 (98/98) | 100,0 (2/2) | 68,1 (98/144) |
| 0,86 | 2,1 (1/48) | 100,0 (98/98) | 100,0 (1/1) | 67,6 (98/145) |

Sensitivity, specificity, PPV and NPV for low physical activity levels during hospitalisation at a selection of consecutive cut-off points of the predicted probability of model 2

PPV = Positive Predictive Value, NPV = Negative Predictive Value

* Patients are considered at high risk of low physical activity levels if their predicted probabilities are at or above this threshold.
** Suggested probability threshold for model 2.
